# Use of anti-androgenic 5α-reductase inhibitors and risk of oesophageal and gastric cancer by histological type and anatomical sub-site

**DOI:** 10.1038/s41416-022-01872-w

**Published:** 2022-06-17

**Authors:** Sirus Rabbani, Giola Santoni, Jesper Lagergren, Shao-Hua Xie

**Affiliations:** 1 Upper Gastrointestinal Surgery, Department of Molecular Medicine and Surgery, Karolinska Institutet, Karolinska University Hospital, Stockholm, Sweden; 2 School of Cancer and Pharmaceutical Sciences, King’s College London, London, UK; 3 School of Public Health and Key Laboratory of Ministry of Education for Gastrointestinal Cancer, Fujian Medical University, Fuzhou, China

**Keywords:** Risk factors, Epidemiology

## Abstract

**Background:**

To investigate if anti-androgenic medications 5α-reductase inhibitors (5-ARIs) decrease the risk of developing oesophageal and gastric tumours, analysed by histological type and anatomical sub-site.

**Methods:**

A Swedish population-based cohort study between 2005 and 2018 where men using 5-ARIs were considered exposed. For each exposed participant, ten male age-matched non-users of 5-ARIs (non-exposed) were included. Multivariable Cox regression provided hazard ratios (HR) with 95% confidence intervals (CI) adjusted for age, calendar year, smoking, non-steroidal anti-inflammatory drugs/aspirin use, and statins use. Further adjustments were made depending on the tumour analysed.

**Results:**

The cohort included 191,156 users of 5-ARIs and 1,911,560 non-users. Overall, the use of 5-ARIs was not associated with any statistically significantly reduced risk of oesophageal or cardia adenocarcinoma (adjusted HR 0.92, 95% CI 0.82–1.02) or gastric non-cardia adenocarcinoma (adjusted HR 0.90, 95% CI 0.80–1.02). However, the use of 5-ARIs indicated a decreased risk of oesophageal or cardia adenocarcinoma among obese or diabetic participants (adjusted HR 0.55, 95% CI 0.39–0.80) and a reduced risk of oesophageal squamous cell carcinoma (adjusted HR 0.49, 95% CI 0.37–0.65).

**Conclusion:**

Users of 5-ARIs may have a decreased risk of developing oesophageal or cardia adenocarcinoma among those obese or diabetic, and a decreased risk of oesophageal squamous cell carcinoma.

## Background

Oesophageal and gastric cancers are the 6th and 2nd leading causes, respectively, of cancer-related deaths globally [1]. The main histological types of oesophageal cancer, i.e. adenocarcinoma and squamous cell carcinoma, have distinct aetiology. Adenocarcinoma is the dominant histological type (>95%) of gastric cancer, but the anatomical sub-sites cardia and non-cardia gastric adenocarcinoma have different aetiology [2, 3]. Oesophageal and cardia adenocarcinoma share the main risk factors of gastro-oesophageal reflux disease and obesity, while tobacco smoking and alcohol overconsumption are the main risk factors for oesophageal squamous cell carcinoma [2]. *Helicobacter pylori* infection is the main risk factor for gastric non-cardia adenocarcinoma, but does not increase the risk of cardia adenocarcinoma, and decreases the risk of oesophageal adenocarcinoma [4].

Oesophageal and cardia adenocarcinoma are characterised by a strong and unexplained male predominance with a 6-to-1 male-to-female ratio in Europe and up to 9-to-1 in North America [5, 6]. The role of sex hormones in the aetiology of these tumours is supported by a 16-year delayed onset in women compared with men, and associations with circulating sex hormone levels [7–10]. Oesophageal squamous cell carcinoma and gastric non-cardia adenocarcinoma are also overrepresented in men, but the male predominance is weaker and is largely explained by sex differences in the prevalence of the main risk factors [4, 11, 12].

The medications 5α-reductase inhibitors (5-ARIs) have anti-androgenic properties and are widely used against benign prostatic hyperplasia. They inhibit the 5α-reductase enzyme and reduce the conversion of testosterone to the more potent androgen dihydrotestosterone [13–15]. The only previous study that has investigated 5-ARIs use and risk of oesophageal and gastric cancer indicated risk reductions, but that study did not separately analyse histological types or sub-sites of these tumours [16].

This study aimed to test the hypothesis that the use of 5-ARIs decreases the risk of oesophageal and gastric cancer, independent of tumour histology and anatomical sub-site, but that the risk is particularly decreased for oesophageal and cardia adenocarcinoma.

## Methods

### Design

This was a nationwide Swedish population-based cohort study of men between July 1, 2005 and December 31, 2018, where exposure to 5-ARIs was examined in relation to the risk of developing the outcomes of oesophageal or gastric cancer. The source population was all male Swedish residents aged 18 years or older who were included in the Swedish Prescribed Drugs and Health Cohort (SPREDH), which has been presented in detail in a cohort description [17]. In brief, SPREDH combines data from four national Swedish health data registries: Prescribed Drug Registry, Patient Registry, Cancer Registry and Cause of Death Registry. These registries achieved complete nationwide coverage well before the study period, and the linkage of individuals’ data between multiple registries was enabled by the unique personal identity number, assigned to each resident in Sweden at birth or immigration. SPREDH included 8,269,978 adults with at least one record of a selected commonly prescribed medication during the study period. The data used for the present study were basic characteristics, prescribed and dispensed medications, healthcare utilisation, diagnoses, and dates and causes of death. Individuals were excluded if they had any previous diagnosis of oesophageal cancer, gastric cancer, or male genital cancers (including prostate cancer), or had undergone oesophagectomy or gastrectomy before cohort entry (details provided in Supplementary Table 1).

### Exposure

The exposure was at least two dispensed records of the 5-ARIs finasteride or dutasteride during the study period. These medications were identified from the Swedish Prescribed Drug Registry by their anatomical therapeutic chemical (ATC) classification codes (Supplementary Table 2). For each exposed participant, ten individuals unexposed to 5-ARIs were randomly selected from SPREDH and matched by age (±1 year) at cohort entry (2nd 5-ARI purchase). Non-users of 5-ARIs were censored if the first dispensation of any 5-ARI was recorded. Non-users were shifted to the exposed group at any second dispensed record of 5-ARIs. All participants were followed up until the date of any of the outcomes, death or the end of the study period (December 31, 2018), whichever occurred first.

### Outcomes

Among a total of three outcomes, the main outcome was oesophageal or cardia adenocarcinoma. These tumours were combined because of their adjacent anatomical positioning, shared aetiology and similarly strong male predominance. The secondary outcomes were oesophageal squamous cell carcinoma and gastric non-cardia adenocarcinoma. All cancer diagnoses were identified from the Cancer Registry by the diagnosis codes in the International Classification of Diseases, 7th version (ICD-7) (150 for oesophageal cancer, 151.1 for cardia cancer, and 151 excluding 151.1 for gastric non-cardia cancer) and histology codes in the WHO/HS/CANC/24 classification (096 for adenocarcinoma and 146 for squamous cell carcinoma).

### Covariates

There were nine covariates in total: age (continuous), calendar year (continuous), gastro-oesophageal reflux disease (yes or no), obesity or diabetes (yes or no), tobacco smoking or smoking-related diagnoses (yes or no), alcohol overconsumption-related diagnoses (yes or no), *Helicobacter pylori* eradication treatment (yes or no), use of non-steroidal anti-inflammatory drugs (NSAIDs) or aspirin (yes or no), and use of statins (yes or no). The combined covariate obesity and diabetes diagnoses were used as a proxy for obesity because the obesity diagnosis was under-recorded in the Patient Registry and the vast majority (81.9%) of diabetes patients are overweight [18]. Diagnoses confirmed within 10 years before cohort entry were searched for in the Patient Registry. *Helicobacter pylori* eradication treatment was defined by a record of a specific eradication package in the Prescribed Drug Registry during the study period and was treated as a time-varying variable. Use of NSAIDs/aspirin and statins were defined by at least two dispensed records of these medications in the Prescribed Drug Registry within one year before cohort entry.

### Statistical analysis

Cox proportional hazards regression was performed to estimate the risks of the study outcomes in 5-ARI users compared with non-users of 5-ARI, providing hazard ratios (HR) with 95% confidence intervals (CI). Both crude and multivariable models were applied. The multivariable models were tailored for each outcome, but all models were adjusted for five covariates: Age, calendar year, tobacco smoking or smoking-related diagnoses, use of NSAIDs/aspirin, and use of statins. In addition to these adjustments, the analyses of oesophageal or cardia adenocarcinoma were adjusted for gastro-oesophageal reflux disease, obesity or diabetes, and *Helicobacter pylori* eradication treatment; analyses of gastric non-cardia adenocarcinoma adjusted for obesity or diabetes and *Helicobacter pylori* eradication treatment; and analyses of oesophageal squamous cell carcinoma adjusted for alcohol overconsumption-related diagnoses. Stratified analyses were performed for oesophageal or cardia adenocarcinoma (main outcome) by age (dichotomised by the median value), gastro-oesophageal reflux disease (yes and no), obesity or diabetes (yes and no), *Helicobacter pylori* eradication treatment (yes and no), tobacco smoking or smoking-related diagnoses (yes and no), and type of 5-ARIs (finasteride and dutasteride). We also tested the possible interaction of the use of 5-ARIs with each of these covariates by adding a multiplicative interaction term in the Cox regression. All models met the proportional-hazard assumption on the basis of Schoenfeld residuals. Two sensitivity analyses were performed to assess the robustness of the results: one excluding those individuals who entered the cohort in 2005 (the first year) and had less than 6 months of drug history, which was done in order to assess new 5-ARI users (incident users); and another that censored individuals who developed any cancer (except for non-malignant melanoma; ICD-7 codes 140-209 excluding 191 in the Cancer Registry) before the outcomes under study.

An experienced biostatistician (GS) followed a detailed study protocol and used the statistical software Stata (Release 16, StataCorp, College Station, TX) when performing the data management and statistical analyses. All analyses were two-sided.

## Results

### Study participants

The study included 191,156 users of 5-ARIs, together accumulating 1,094,334 exposed person-years at risk. These were compared with 1,911,560 non-users of 5-ARIs who contributed to a total of 9,874,357 unexposed person-years. The mean duration of 5-ARI use was 3.8 years (interquartile range 0.61–6.11 years). The two groups had similar characteristics, except for a slightly higher prevalence of gastro-oesophageal reflux disease and *Helicobacter pylori* eradication treatment and a lower prevalence of alcohol overconsumption-related diagnoses in the 5-ARIs users (Table 1). Among users of 5-ARIs 33.7% had no record of benign prostatic hyperplasia diagnosis compared to 78.9% in the non-user group (Supplementary Table 4).

**Table 1:** Characteristics of the study participants by use of 5α-reductase inhibitors.

Characteristics	Users number (%)	Non-users number (%)
Total	191,156 (100.0)	1,911,560 (100.0)
Age at entry, in years		
≤59	16,191 (8.5)	163,168 (8.5)
60–69	53,745 (28.1)	540,396 (28.3)
70–79	72,312 (37.8)	721,023 (37.7)
≥80	48,908 (25.6)	486,973 (25.5)
Mean (± standard deviation)	72.5 (±10.6)	72.4 (±10.6)
Cohort entry		
July 2005–June 2006	31,942 (16.7)	319,420 (16.7)
July 2006−2012	78,947 (41.3)	789,470 (41.3)
2013−2018	80,267 (42.0)	802,670 (42.0)
*Helicobacter pylori* treatment		
Before or at cohort entry	3452 (1.8)	28,917 (1.5)
After cohort entry	3036 (1.6)	24,356 (1.3)
Use of NSAIDs/aspirin	68,380 (35.8)	668,502 (35.0)
Use of statins	55,803 (29.2)	560,233 (29.3)
Gastro-oesophageal reflux disease	12,358 (6.5)	107,481 (5.6)
Tobacco smoking or smoking-related diagnoses	9959 (5.2)	98,371 (5.2)
Alcohol overconsumption-related diagnoses	3704 (1.9)	53,960 (2.8)
Obesity or diabetes	24,820 (13.0)	267,722 (14.0)
Deaths during follow-up	55,079 (28.8)	540,195 (28.3)

*NSAIDs* non-steroidal anti-inflammatory drugs.

### Risk of oesophageal or cardia adenocarcinoma

A total of 345 users of 5-ARIs and 3328 non-users were diagnosed with oesophageal or cardia adenocarcinoma during the follow-up. The use of 5-ARIs was associated with a slightly decreased point estimate of oesophageal or cardia adenocarcinoma, although the association was not statistically significant (adjusted HR 0.92, 95% CI 0.82–1.02) (Table 2). In the stratified analyses, the use of 5-ARIs was associated with decreased HRs in participants with obesity or diabetes (adjusted HR 0.55, 95% CI 0.39–0.80). No such statistically significant associations were found in the stratified analyses by age, gastro-oesophageal reflux disease, *Helicobacter pylori* treatment, or tobacco smoking or smoking-related diagnoses, although all point estimates were below 1 (Table 3). The point estimates were similar for users of finasteride only (adjusted HR 0.91, 95% CI 0.81–1.03) and dutasteride only (adjusted HR 0.95, 95% CI 0.73–1.24) (Table 4).

**Table 2:** Use of 5α-reductase inhibitors and risk of oesophageal and gastric cancer by histological type and anatomical sub-site.

Outcome	Number of cases in users/non-users	Crude hazard ratio (95% confidence interval)	Adjusted hazard ratio (95% confidence interval)*
Oesophageal or cardia adenocarcinoma			
Non-users	3328	1.00 (Reference)	1.00 (Reference)
Users	345	0.93 (0.83–1.04)	0.92 (0.82–1.02)
Oesophageal squamous cell carcinoma			
Non-users	985	1.00 (Reference)	1.00 (Reference)
Users	53	0.48 (0.37–0.64)	0.49 (0.37–0.65)
Non-cardia gastric adenocarcinoma			
Non-users	2567	1.00 (Reference)	1.00 (Reference)
Users	264	0.93 (0.82–1.06)	0.90 (0.80–1.02)

^*^Adjusted for age, calendar year, tobacco smoking or smoking-related diagnoses, use of non-steroidal anti-inflammatory drugs or aspirin, and use of statins, with further adjustment for gastro-oesophageal reflux disease, obesity or diabetes, and *Helicobacter pylori* treatment for oesophageal or gastric cardia adenocarcinoma, further adjustment for obesity or diabetes, and *Helicobacter pylori* treatment for gastric non-cardia adenocarcinoma, and further adjustment for alcohol overconsumption-related diagnoses for oesophageal squamous cell carcinoma.

**Table 3:** Associations between use of 5α-reductase inhibitors and risk of oesophageal or cardia adenocarcinoma in stratified analyses.

Covariates	Number of cases in users/non-users	Adjusted hazard ratio (95% confidence interval)^*^	Interaction *P* value
Age, years			
<73	151/1627	0.85 (0.73–1.01)	
≥73	194/1701	0.98 (0.85–1.14)	0.216
Gastro-oesophageal reflux disease			
No	312/3030	0.92 (0.82–1.04)	
Yes	33/298	0.86 (0.60–1.24)	0.734
Obese or diabetes			
No	314/2766	0.97 (0.87–1.10)	
Yes	31/562	0.55 (0.39–0.80)	0.004
*Helicobacter pylori* treatment			
No	335/3212	0.93 (0.83–1.04)	
Yes	10/116	0.65 (0.34–1.24)	0.286
Tobacco smoking or smoking-related diagnoses			
No	326/3088	0.93 (0.83–1.05)	
Yes	19/240	0.69 (0.43–1.09)	0.115

^*^Adjusted for the calendar year, use of non-steroidal anti-inflammatory drugs or aspirin, use of statins, and all the other variables presented hereby.

**Table 4:** Associations between use of different types of 5α-reductase inhibitors and risk of oesophageal or cardia adenocarcinoma.

Use of 5α-reductase inhibitors	Number of cases	Crude hazard ratio (95% confidence interval)	Adjusted hazard ratio (95% confidence interval)^*^
Non-users	3328	1.00 (Reference)	1.00 (Reference)
Finasteride only	284	0.93 (0.82–1.05)	0.91 (0.81–1.03)
Dutasteride only	57	0.93 (0.71–1.21)	0.95 (0.73–1.24)

^*^Adjusted for age, calendar year, gastro-oesophageal reflux disease, obesity or diabetes, *Helicobacter pylori* treatment, tobacco smoking or smoking-related diagnoses, use of non-steroidal anti-inflammatory drugs or aspirin, and use of statins.

### Risk of oesophageal squamous cell carcinoma

The risk of oesophageal squamous cell carcinoma was lower in users of 5-ARIs than in non-users (adjusted HR 0.49, 95% CI 0.37–0.65) (Table 2).

### Risk of gastric non-cardia adenocarcinoma

The use of 5-ARIs was associated with a slightly decreased point estimate of gastric non-cardia adenocarcinoma, but this was not statistically significant (adjusted HR 0.90, 95% CI 0.80–1.02) (Table 2).

### Sensitivity analyses

The HRs for all outcomes remained virtually unchanged in the sensitivity analyses excluding participants entering the cohort in 2005 and without at least 6 months of drug history and also in analyses with censoring of participants who developed any cancer during follow-up (Table 5).

**Table 5:** Sensitivity analyses of use of 5α-reductase inhibitors and risk of oesophageal and gastric cancer by histological type and anatomical sub-site.

Outcomes	Number of cases in users/non-users	Adjusted hazard ratio (95% confidence interval)^*^
*Excluding participants with <1 year of follow-up*		
Oesophageal or gastric cardia adenocarcinoma		
Non-user	2385	1.00 (Reference)
User	247	0.94 (0.82–1.07)
Gastric non-cardia adenocarcinoma		
Non-user	1707	1.00 (Reference)
User	182	0.96 (0.82–1.12)
Oesophageal squamous cell carcinoma		
Non-user	684	1.00 (Reference)
User	40	0.55 (0.40–0.76)
*Censoring those diagnosed with other cancers during follow-up*		
Oesophageal or gastric cardia adenocarcinoma		
Non-user	3328	1.00 (Reference)
User	345	0.94 (0.84–1.05)
Gastric non-cardia adenocarcinoma		
Non-user	2567	1.00 (Reference)
User	264	0.92 (0.81–1.05)
Oesophageal squamous cell carcinoma		
Non-user	985	1.00 (Reference)
User	53	0.50 (0.38–0.67)

^*^Adjusted for age, calendar year, tobacco smoking or smoking-related diagnoses, use of non-steroidal anti-inflammatory drugs or aspirin, and use of statins, with further adjustment for gastro-oesophageal reflux disease, obesity or diabetes, and *Helicobacter pylori* treatment for oesophageal or cardia gastric adenocarcinoma, further adjustment for obesity or diabetes, and *Helicobacter pylori* treatment for non-cardia gastric adenocarcinoma, and further adjustment for alcohol overconsumption-related diagnoses for oesophageal squamous cell carcinoma.

## Discussion

This study suggests a decreased risk of oesophageal or cardia adenocarcinoma in men using 5-ARIs with obesity or diabetes diagnoses. The use of 5-ARIs was also associated with a decreased risk of oesophageal squamous cell carcinoma.

Among the strengths of the study are the population-based design, the complete follow-up, and high-quality and prospectively collected data that allowed adjustment for several covariates and stratified analyses. Confounding remains a limitation of this observational study. However, confounding by indication should not be a major issue because 5-ARIs and their indication of prostatic hyperplasia are not associated with the factors associated with any of the studied tumours. Moreover, the results were adjusted for all main risk factors, which did not influence the risk estimates. Yet, due to the lack of detailed data on several risk factors for oesophageal or gastric cancer, e.g. severity of gastro-oesophageal reflux disease and duration and intensity of tobacco smoking, residual confounding cannot be dismissed. Because we used the combined covariate of obesity and diabetes as a proxy for the under-recorded obesity data, we were not able to examine the specific interaction of 5-ARIs use with obesity and/or diabetes. Due to the inherent time-related bias in observational pharmaco-epidemiological studies, particularly because the duration of use or accumulated exposure closely correlate with the follow-up, we did not assess the duration of 5-ARIs use in relation to the risk of oesophageal or gastric cancer. Despite the large size of the cohort, another limitation was a limited statistical power in some of the analyses.

To our knowledge, only one previous study has investigated the associations between 5-ARIs use and the risk of oesophageal and gastric cancer. A Scottish nested case-control study found a reduced overall risk of oesophageal and gastric cancer among users of the 5-ARI *finasteride* (adjusted OR 0.68, 95% CI 0.50–0.94), but no association remained when oesophageal cancer and gastric cancer were analysed separately [16]. That study lacked data on histological types of the tumours and made no distinction between the sub-sites cardia and non-cardia gastric cancer. In contrast, the present study separately analysed histological types and anatomical sub-sites, which is warranted considering the major differences in aetiology and strengths of the male predominance between these tumours.

An obvious decreased risk of oesophageal or cardia adenocarcinoma was observed in 5-ARI users with obesity or diabetes. This finding suggests interactions between obesity and sex hormonal exposures. Obesity is a well-established risk factor for these tumours and obesity decreases circulating levels of testosterone and dihydrotestosterone [19–21]. As indicated by two recent prospective studies, low testosterone levels may increase the risk of oesophageal and cardia adenocarcinoma [9, 10]. Because 5-ARIs decrease levels of dihydrotestosterone with a compensatory effect leading to increased testosterone levels, it is possible that the obese individuals with lowered testosterone levels are more likely to benefit from the potential protective anti-carcinogenic effect of normal or elevated testosterone levels. Yet, the mechanisms are unclear, especially for dihydrotestosterone which is more potent than testosterone in binding to androgen receptors. However, this has not been associated with a risk of oesophageal or cardia adenocarcinoma [10].

A substantially reduced risk of oesophageal squamous cell carcinoma was observed in 5-ARIs users. It has been suggested that sex hormonal factors may play a role also in the aetiology of this tumour. Several studies have reported the expression of androgen receptors in oesophageal squamous cell carcinoma tissue and in vitro studies have shown that testosterone can induce the proliferation of oesophageal squamous cell carcinoma cell lines [22–25]. However, the epidemiologic evidence regarding associations between sex hormonal exposures and oesophageal squamous cell carcinoma is limited and inconclusive [26–31]. The present study indicates the role of anti-androgenic exposure in the aetiology of this cancer.

The results also suggested a weak association between the use of 5-ARIs and the risk of gastric non-cardia adenocarcinoma, although this was not statistically significant. Pre-clinical studies have indicated a limited role of sex hormones in gastric carcinogenesis [32–34], and the results of the current study do not support any strong risk reduction associated with 5-ARI use.

In conclusion, this population-based cohort study indicates that 5-ARI use is associated with a decreased risk of oesophageal or cardia adenocarcinoma in individuals with obesity or diabetes and a decreased risk of oesophageal squamous cell carcinoma, while no clear risk reduction was found for gastric non-cardia adenocarcinoma. The available evidence is currently too limited to support any changes in clinical practice or prompt any randomised clinical trials, but more large cohort studies examining the associations between 5-ARI use and the risk of oesophageal and gastric cancer are warranted.

## Supplementary information

Supplemental material

## Data Availability

Data supporting the results are available upon request.
