# Supplementary material for: Use of anti-androgenic 5α-reductase inhibitors and risk of oesophageal and gastric cancer by histological type and anatomical sub-site
Source: Br J Cancer. 2022 Jun 17;127(5):892–7. doi: 10.1038/s41416-022-01872-w (PMC9427733; doi:10.1038/s41416-022-01872-w)
Supplement: Supplementary file 1 — Supplemental material [file 41416_2022_1872_MOESM1_ESM.docx]

Codes used to identify the exclusion criteria, 5-ARI medication types as well as covariates from the appropriate registries.

| **Supplementary Table 1.** Diagnosis and surgical procedure codes for the exclusion criteria | | | | |
| --- | --- | --- | --- | --- |
| Exclusion criteria |  | Data source |  | Code |
| (1) Previous diagnosis of the study outcome |  | Cancer Registry |  | Main outcome **-** Oesophageal or gastric cardia adenocarcinoma (ICD-7 code 150 or 151.1 and histology code 096)  Comparison outcome 1 – Gastric non-cardia adenocarcinoma (ICD-7 code 151 exclusion of 151.1 and histology code 096)  Comparison outcome 2 – Oesophageal squamous cell carcinoma (ICD-7 code 150 and histology code 146) |
| (2) Previous testis, prostate or other male genital cancer |  | Cancer Registry |  | ICD-7 codes 177-179 |
| (3) Previous oesophagectomy or gastrectomy |  | Patient Registry |  | 2820, 2821, 2822, 2829, 4411-4420, 4422, 4424-4426, 4429, 4430, 4432, 4434, or 4435 before 1997 and JCC, JDC or JDD after 1997. |
| ICD-7: International Classification of Diseases, 7^th^ version. | | | | |

| **Supplementary Table 2.** Anatomical therapeutic chemical (ATC) classification codes for 5α-reductase inhibitors | | |  |
| --- | --- | --- | --- |
| Type |  | Code |  |
| Finasteride |  | D11AX10 and G04CB01 |  |
| Finasteride combined with alfuzosin |  | G04CA51 |  |
| Dutasteride |  | G04CB02 |  |
| Dutasteride combined with tamsulosin |  | G04CA52 |  |
|  | | | |

| **Supplementary Table 3.** Diagnosis codes according to the International Classification of Diseases (ICD) and anatomical therapeutic chemical (ATC) classification codes for covariates | | | | |
| --- | --- | --- | --- | --- |
| Covariates |  | Codes | | |
|  |  | ICD-9-SE |  | ICD-10-SE |
| Reflux |  | 787B, 530B, 530C, 553D |  | R12, K20, K21, K44 |
| Obesity |  | 278A |  | E66 |
| Diabetes |  | 250 |  | E10-E14 |
| Tobacco |  | 490, 491, 492, 494, 496 Chronic obstructive pulmonary disease related diagnoses  305B Tobacco overconsumption; |  | Z72.0 Tobacco use;  J40-J44, J47 Chronic obstructive pulmonary disease related diagnosis  F17 Psychiatric and addiction related diagnoses caused by tobacco consumption |
| Alcohol overconsumption |  | 291, 303, V79B Alcohol-related disorders;  305A Degeneration of nervous system due to alcohol;  357F Alcohol polyneuropathy;  359E Alcoholic myopathy;  425F Alcohol cardiomyopathy;  535D Alcoholic gastritis;  571, 572W Alcoholic liver disease;  577B Alcohol-induced chronic pancreatitis;  V65E Alcohol abuse counselling and surveillance;  790D Increased alcohol serum levels  977D overdose using drugs used to support treatment of chronic alcoholism |  | F10 Alcohol-related disorders;  G31.2 Degeneration of nervous system due to alcohol;  G62.1 Alcohol polyneuropathy;  G72.1 Alcoholic myopathy;  I42.6 Alcohol cardiomyopathy;  K29.2 Alcoholic gastritis;  K70 Alcoholic liver disease;  K86.0 Alcohol-induced chronic pancreatitis;  Z71.4 Alcohol abuse counselling and surveillance;  T51.0, T51.1, T51.9 Alcohol poisoning |
|  |  | ATC codes | | |
| *Helicobacter pylori* treatment |  | A02BD | | |
| Non-steroidal anti-inflammatory drugs or aspirin |  | M01A, N02BA, B01AC06  C10BX01, C10BX02, C10BX04, C10BX05, C10BX06, C10BX08 C10BX12, C07FX02, C07FX03, C07FX04 | | |
| Statins |  | C10AA, C10B | | |
| ICD-9-SE: Swedish version of International Classificiation of Diseases, 9^th^ revision; ICD-10-SE: Swedish version of International Classificiation of Diseases, 10^th^ revision | | | | |

| **Supplementary Table 4.** Frequency of prostatic hyperplasia diagnosis in users and non-users of 5-ARI | | | |  |
| --- | --- | --- | --- | --- |
|  | Users of 5-ARI  Number (%) |  | Non-users of 5-ARI  Number (%) |  |
| No diagnosis | 64,456 (33.7) |  | 1,508,419 (78.9) |  |
| Diagnosis before study inclusion | 94,083 (49.2) |  | 240,440 (12.6) |  |
| Diagnosis before after inclusion | 32,617 (17.1) |  | 162,701 (8.5) |  |
